# Supplementary material for: Site-selective chemical reactions by on-water surface sequential assembly
Source: Nat Commun. 2023 Dec 14;14:8313. doi: 10.1038/s41467-023-44129-7 (PMC10721922; doi:10.1038/s41467-023-44129-7)
Supplement: Supplementary file 1 — Supplementary Information [file 41467_2023_44129_MOESM1_ESM.pdf]

Site-selective chemical reactions by on-water surface sequential assembly

Anupam Prasoon[1,2,+], Xiaoqing Yu[3,+], Mike Hambsch[4], David Bodesheim[5], Kejun Liu[1], Angelica Zacarias[2], Nguyen Ngan Nguyen[1], Takakazu Seki[3], Aerzoo Dianat[5], Alexander Croy[6], Gianaurelio Cuniberti[5,7], Philippe Fontaine[8], Yuki Nagata[3], Stefan C. B. Mannsfeld[4]*, Renhao Dong[1,9]*, Mischa Bonn[3]*, Xinliang Feng[1,2]*

[1]Center for Advancing Electronics Dresden (cfaed) and Faculty of Chemistry and Food Chemistry, Technische Universität Dresden, 01062 Dresden, Germany

[2]Max Planck Institute for Microstructure Physics, Halle (Saale) D-06120, Germany

[3]Max Planck Institute for Polymer Research, Ackermannweg 10, 55128 Mainz, Germany

[4]Center for Advancing Electronics Dresden (cfaed) and Faculty of Electrical and Computer Engineering, Technische Universität Dresden, 01062 Dresden, Germany

[5]Institute for Materials Science and Max Bergmann Center of Biomaterials, Technische Universität Dresden, 01062 Dresden, Germany

[6]Institute of Physical Chemistry, Friedrich Schiller University Jena, 07737 Jena, Germany

[7]Dresden Center for Computational Materials Science (DCMS), Technische Universität Dresden, 01062 Dresden, Germany

[8]Synchrotron SOLEIL, L'Orme des Merisiers, Départementale 128, 91190, Saint-Aubin, France

[9]Key Laboratory of Colloid and Interface Chemistry of the Ministry of Education, School of Chemistry and Chemical Engineering, Shandong University, Jinan, China.

[+]These authors contributed equally: Anupam Prasoon, Xiaoqing Yu

[*]E-mail: stefan.mannsfeld@tu-dresden.de; renhaodong@sdu.edu.cn; bonn@mpip-mainz.mpg.de; xinliang.feng@tu-dresden.de

Table of Contents

Materials..4

Supplementary Fig. 1..5

Supplementary Fig. 2…..6

Supplementary Fig. 3…..7

Supplementary Fig. 4…..8

Supplementary Fig. 5…..9

Supplementary Fig. 6…..10

Supplementary Tab. 1..11

Supplementary Fig. 7…..12

Supplementary Fig. 8…..13-15

Supplementary Fig. 9…..16

Supplementary Tab. 2…..17

Supplementary Fig. 10…..18

Supplementary Fig. 11…..19-20

Supplementary Fig. 12…..21-22

Supplementary Fig. 13…..23

Supplementary Fig. 14…..24

Supplementary Fig. 15…..25

Supplementary Fig. 16, 17…..26

Supplementary Fig. 18, 19…..27-28

Supplementary Fig. 20…..29

Supplementary Fig. 21…..30

Supplementary Fig. 22…..31-32

Supplementary Fig. 23…..33

Supplementary Fig. 24…..34

Supplementary Fig. 25…..35

Supplementary Fig. 26…..36

Supplementary Fig. 27…..37

References...38-39

Materials

The chemicals listed (e.g. 5-(4-aminophenyl)-10,15,20-(triphenyl)porphyrin (**R1**) , anthra[2,1,9-def:6,5,10-d'e'f']diisochromene-1,3,8,10-tetraone (**R2**), 2,5-dihydroxyterephthalaldehyde (**R3**), 5-(4-carboxyphenyl)-10,15,20-(triphenyl)porphyrin (**R4**), 1,2,4,5-benzenetetramine tetrahydrochloride (**R5**), sodium oleyl sulfate (SOS), hexadecyltrimethylammonium bromide (CTAB) and solvents (e.g. chloroform) were obtained from PorphyChem, abcr GmbH, and Sigma-Aldrich and used without further purification. Purified water was obtained through a Milli-Q purification system (Merck KGaA). All the site-selective chemical reactions were carried out under ambient atmospheric conditions. The substrates used (e.g., 300 nm SiO_2/Si wafer, quartz glass, and copper grids) were obtained from Microchemicals and Plano GmbH.

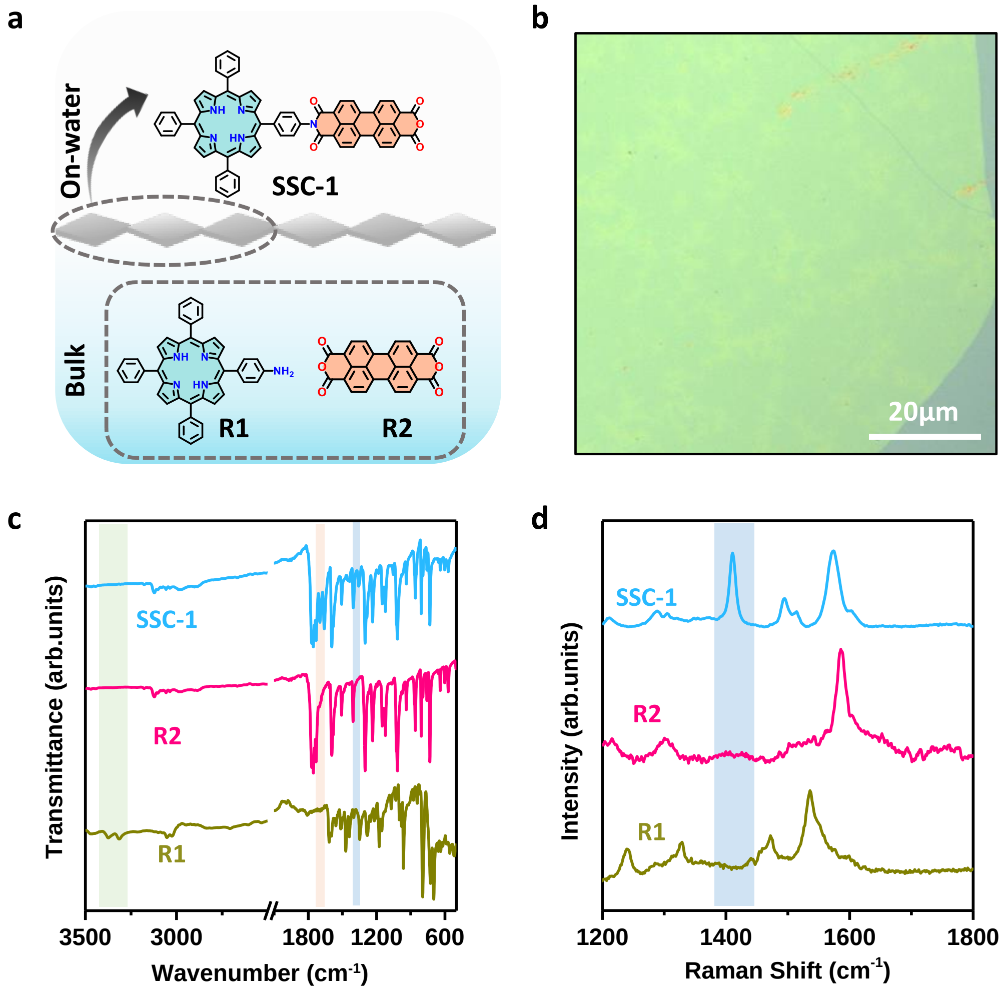

Supplementary Fig. 1. Site-selective programmable chemical reaction SSR-1 on the water surface. (a) Schematic of experimentally observed site-selective compound, **SSC-1** on the water surface. (b) An optical microscope image of the **SSC-1** film on a SiO_2/Si substrate. (c) ATR-FTIR spectra of **R1**, **R2** and **SSC-1**. ATR-FTIR spectroscopy of **SSC-1** shows the appearance of the imide C–N bond at ~1353 cm^{-1} and imide *C*=*O* bond at ~1690 cm^{-1}, as well as the complete vanishing of the N-H stretch at ~3320 cm^{-1} from **R1**. (d) Raman spectra of **R1**, **R2,** and **SSC-1**. A new peak at ~1408 cm^{-1} appears in the **SSC-1** spectra, revealing the formation of an imide C-N bond.

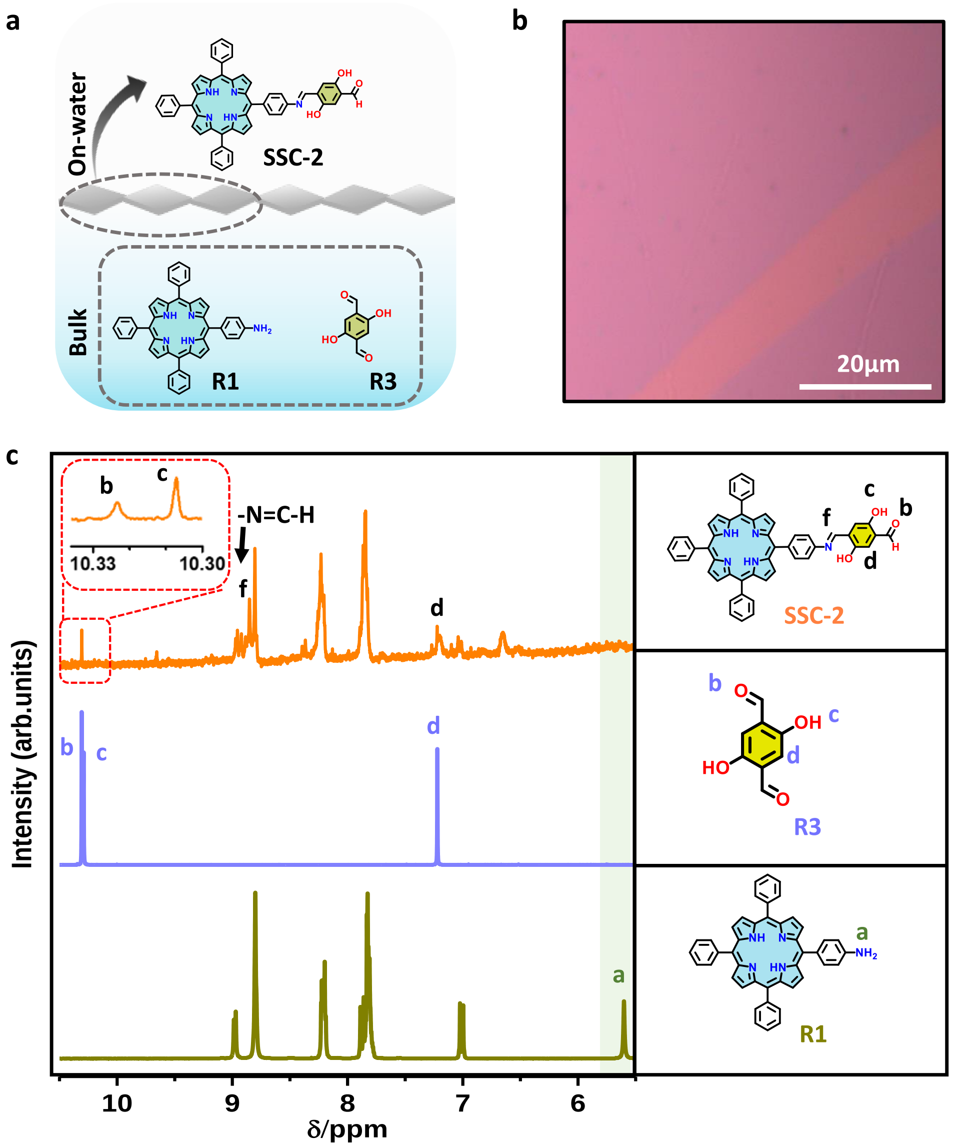

Supplementary Fig. 2. Site-selective programmable chemical reaction SSR-2 on the water surface. (a) Schematic of experimentally observed site-selective compound, **SSC-2** on the water surface. (b) An optical microscope image of the SSC-2 film on a SiO_2/Si substrate. (c) ^{1}H NMR spectra of **R1**, **R3** and **SSC-2** in DMSO-d_6. (Note: For the **SSC-2** NMR measurement, we performed 54 reactions and collected the water surface film from each of the 54 reaction beakers.)

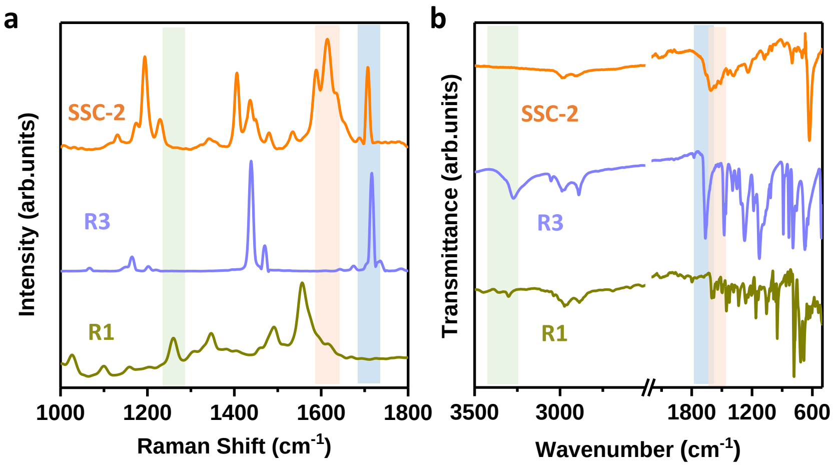

Supplementary Fig. 3. Spectroscopic characterizations of R1, R3 and SSC-2. (a) Raman spectra of **R1**, **R3** and **SSC-2**. In contrast to the **R1** and **R3**, a new band at ~1608 cm^{-1} characteristic for –C=N stretching is observed for **SSC-2**, highlighting the formation of imine bond and also with the appearance of C=O aldehyde bond of R3 showing site-selective bond formation. In **SSC-2**, the NH_2 band at ~1266 cm^{-1} completely vanishes from **R1**. (b) ATR-FTIR spectroscopy of **SSC-2** shows the appearance of the –C=N bond at ~1612 cm^{-1} and imide *C*=*O* bond at ~1670 cm^{-1}, as well as the complete vanishing of the N-H stretch at ~3305 cm^{-1} from **R1**.

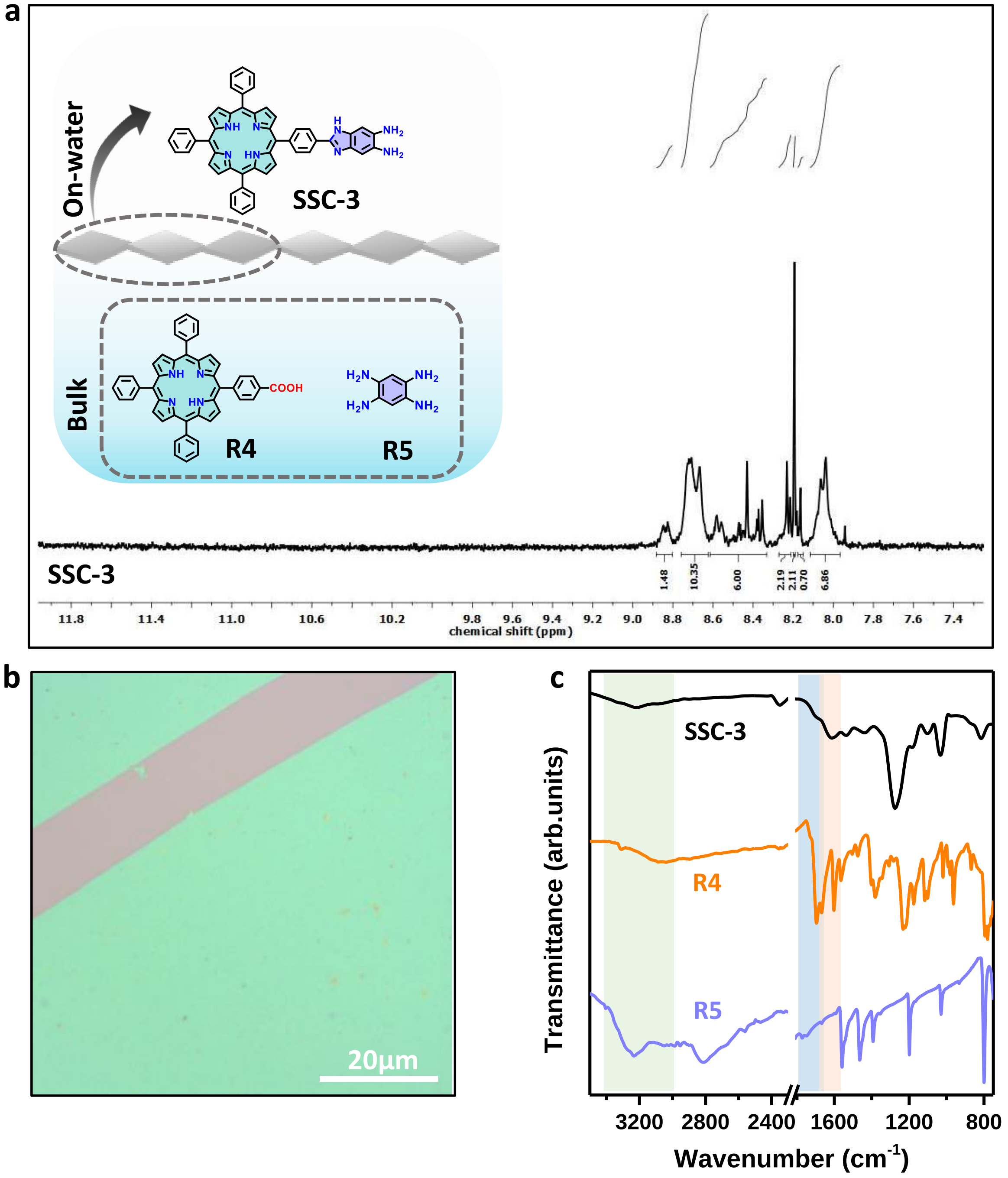

Supplementary Fig. 4. Site-selective programmable chemical reaction SSR-3 on the water surface. (a) Schematic of experimentally observed site-selective compound, **SSC-3** on the water surface and ^{1}H NMR spectra **SSC-3** in DMSO-d_6. (Note: For the **SSC-3** NMR measurement, we performed 48 reactions and collected the water surface film from each of the 48 reaction beakers.) (b) An optical microscope image of the **SSC-3** film on a SiO_2/Si substrate. (c) ATR-FTIR spectra of **R4**, **R5** and **SSC-3**. ATR-FTIR spectroscopy of **SSC-3** shows, the presence of N-H str. at 3328 cm^{-1}, C=N benzimidazole ring skeleton at 1625 cm^{-1} and absence of C=O at 1705 cm^{-1} band in **SSC-3**.

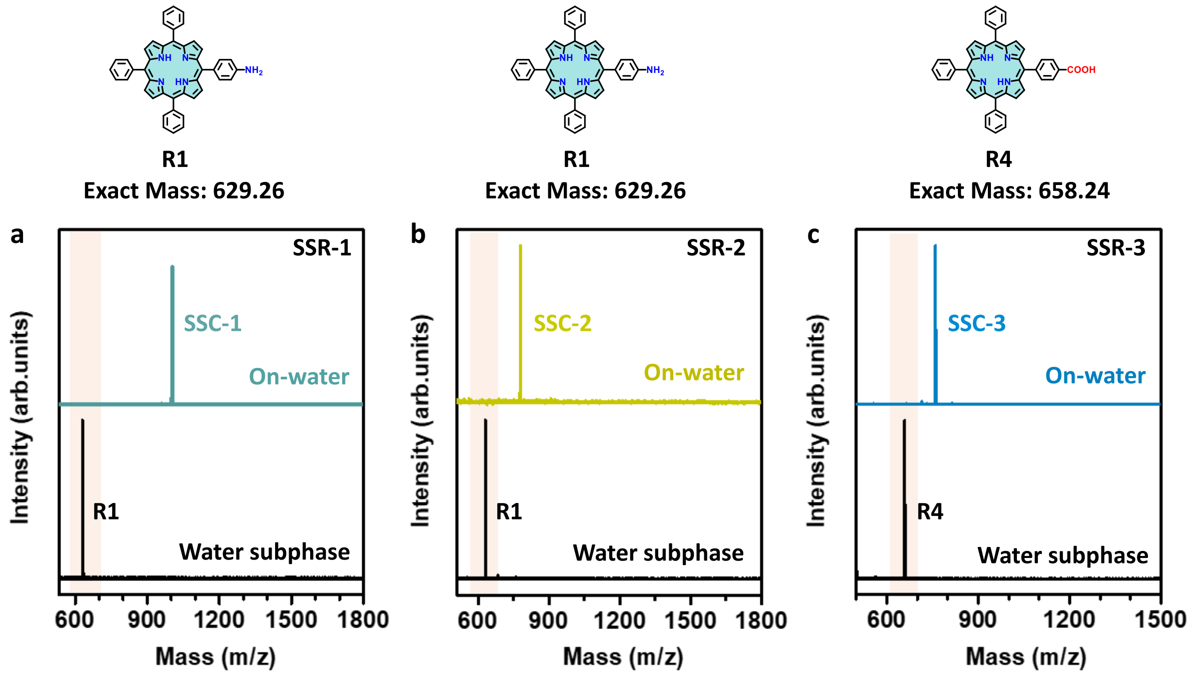

Supplementary Fig. 5. MALDI-TOF MS analysis of the (a) **SSC-1**, (b) **SSC-2** and (c) **SSC-3** synthesized on the water surface and in the water subphase. The overall reaction was realized to be suppressed in the water subphase.

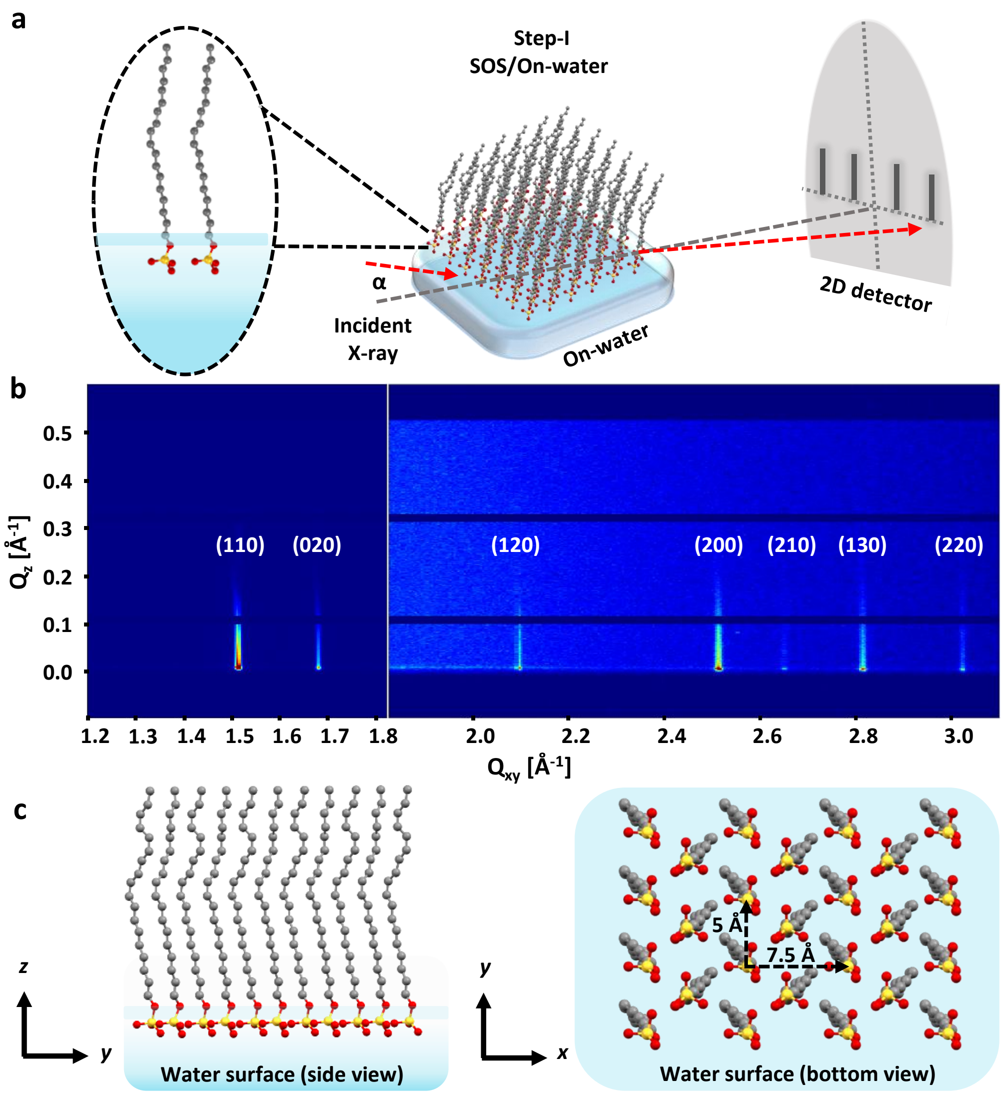

Supplementary Fig. 6. In-situ Grazing Incidence X-ray Diffraction (GIXD) on the water surface. (a) Schematic representation of the in-situ GIXD measurement setup directly on the water surface, which is very sensitive towards the in-plane molecular structure of monolayers.. Therefore, in-situ step-by-step GIXD was performed directly on the water surface, which commences from step-I, i.e., the surfactant monolayer formation. Surfactants are amphiphilic molecules with a long hydrocarbon tail and a polar head group, which adsorb at the air-water interface with the hydrophilic heads towards the water surface and the hydrophobic tails towards the air. (b) The corresponding measured GIXD scattering profile of SOS surfactant exhibited distinct and sharp diffraction peaks which correspond to a unit cell with a = 5 Å, b = 7.48 Å, and γ = 89.96°. Simulated structure of SOS surfactant monolayer on water surface (c) side view (d) bottom view.

Peak	(hk)	Q_{xy} [Å^{-1}]	d_{xy} [Å]
a	(11)	1.51	4.15
b	(02)	1.68	3.74
c	(12)	2.10	2.99
d	(20)	2.51	2.50
e	(21)	2.65	2.37
f	(13)	2.82	2.23
g	(22)	3.02	2.08

Supplementary Tab. 1. The corresponding measured GIXD scattering profile of SOS surfactant monolayer exhibited seven distinct and sharp diffraction peaks along with d_{xy} [Å] values.

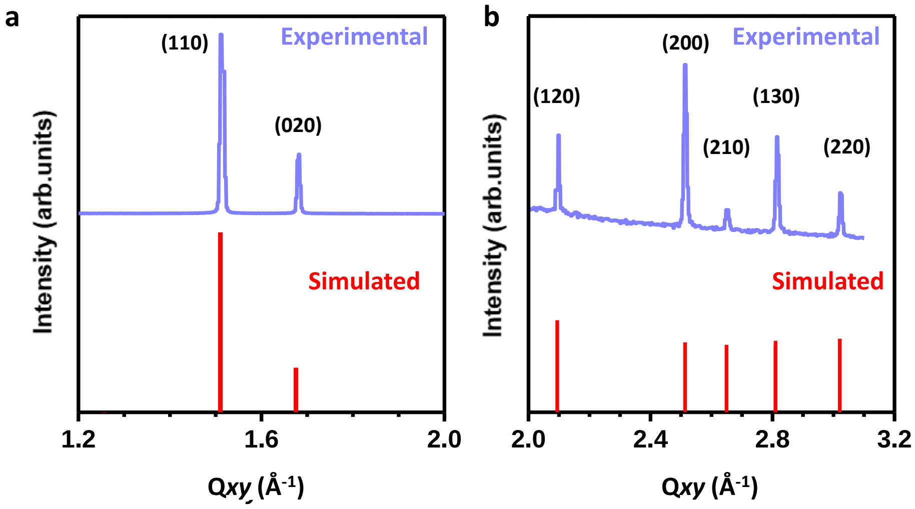

Supplementary Fig. 7. Experimental and simulated GIXD diffraction in-plane peaks (near $Q_z = 0$, Q represents scattering vector) projections (a) lower (1.2 - 2.0) Q_{xy} (b) higher (2.0 - 3.2) Q_{xy}.

GIXD Simulation of SOS monolayer

In order to calculate the reflexes and the intensities of the GIXD experiment, we approximated that scattering only occur in the xy-plane, meaning we only simulate reflections of the in-plane components scattering vector:

$$Q_{xy} = \left| h\,\overrightarrow{b_1} + k\,\overrightarrow{b_2} \right|$$

With $\overrightarrow{b_1}$ and $\overrightarrow{b_2}$ as the reciprocal lattice vectors and h and k as the Miller-indices

The 2D structure factor is calculated as:

$$\mathrm{F_{hk}} = \sum_{\mathrm{i}} \mathrm{f_i}\ \mathrm{e}^{-2\mathrm{i}\pi(\mathrm{h}\cdot\mathrm{x_i}+\mathrm{k}\cdot\mathrm{y_i})}$$

With x_i and y_i as the fractional coordinates and $\mathrm{f_i}$ as the atomic form factor of the atom i.

The $\mathrm{f_i}$ is approximated by a sum of Gaussians:

$$\mathrm{f_i}(\,\mathrm{Q_{xy}}) \;=\; \sum_{\mathrm{i}=1}^{4} \mathrm{a_i} \mathrm{e}^{\left(-\mathrm{b_i}\left(\frac{\mathrm{Q_{xy}}}{4\pi}\right)^2\right)} \;+\; \mathrm{c}$$

where $\mathrm{a_i}$, $\mathrm{b_i}$ and c are tabulated parameters for the respective atom type[1].

Since multiple h and k combinations can result in the same Q_{xy} value, a total structure factor $\mathrm{F}(\mathrm{Q_{xy}})$ was estimated by summing over multiple h and k combinations in the range of -4 to to 4:

$$F(\mathrm{Q_{xy}}) = \sum_{h=-4}^{h=4} \sum_{k=-4}^{k=4} F_{hk}$$

The multiplication of the total structure factor with its complex conjugate yields the scattering intensity:

$$\mathrm{I}(\mathrm{Q_{xy}}) = \mathrm{F}(\mathrm{Q_{xy}})\mathrm{F}(\mathrm{Q_{xy}})^{*}$$

In order to solve the structure, first, a simple 2D cell with a one-site basis was assumed and the cell parameters were screened until a good fit with the experimental reflexes was obtained. For this simple model, a constant atomic form factor was chosen.

The parameters of a=b=4.52 Å and γ=112.6° yielded the best result, as shown in (Supplementary Figs. 8a, b). Many of the reflexes are a perfect match. However, the reflexes at 2.08 $Å^{-1}$ and 2.63 $Å^{-1}$ are missing.

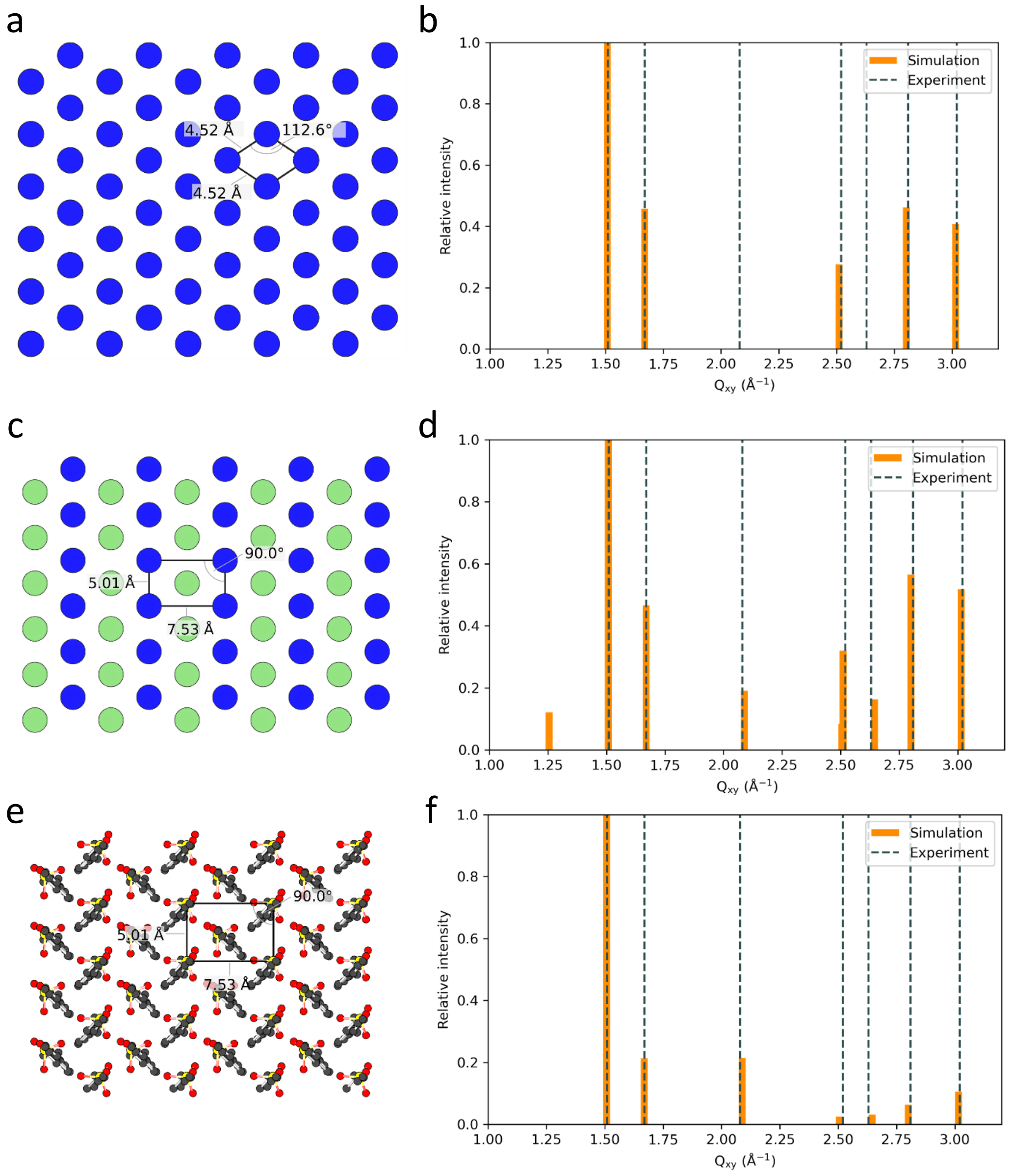

Supplementary Fig. 8. (a) Structure of the simple one-site basis lattice. (b) Corresponding simulated diffraction pattern including the reflex positions of the experiment as dashed line. (c) Structure of two-site basis centered rectangular lattice. (d) Corresponding simulated diffraction pattern including the reflex positions of the experiment as dashed line. (e) Structure of two-site basis centered rectangular

lattice. (f) Corresponding simulated diffraction pattern including the reflex positions of the experiment as dashed line.

This can be resolved, if a two-site basis is introduced. This is achieved by introducing a bigger unit-cell which is based on a centered rectangular lattice and includes now two distinguishable sites. This new unit-cell has the dimensions a=7.53 Å, b=5.01 Å and γ=90° and site A is at fractional coordinates (0,0) and site B is at (0.5, 0.5), as shown in (Supplementary Fig. 8c) . The two sites must be distinguishable and therefore, they need to have two different atomic form factors. Hence, for site A a dummy carbon atom and for site B a dummy sulfur atom was chosen as distinguishable sites. This results in the simulated reflex pattern in (Supplementary Fig. 8d) where the positions are in very good agreement with the experimental peak positions, except for a reflex at 1.26 $Å^{-1}$.

For the packing pattern of SOS, this means that it must be packed in a way that there are two distinguishable sites. This is achieved by a so-called herringbone pattern, where the SOS on the second site is rotated by 90° around the z-axis, as shown in (Supplementary Fig. 8e). In the resulting diffraction pattern, the peak at 1.26 $Å^{-1}$, which corresponds to the (01) reflex, disappears due to symmetry, as shown in (Supplementary Fig. 8f). The herringbone packing with cell parameters of a=7.5 Å, b=5.0 Å and γ=90° is also known from fatty acid monolayers and the experimental and simulated pattern agrees with literature[2].

The code for the analysis can be found at https://github.com/DBodesheim/2D_Scattering_Analysis/

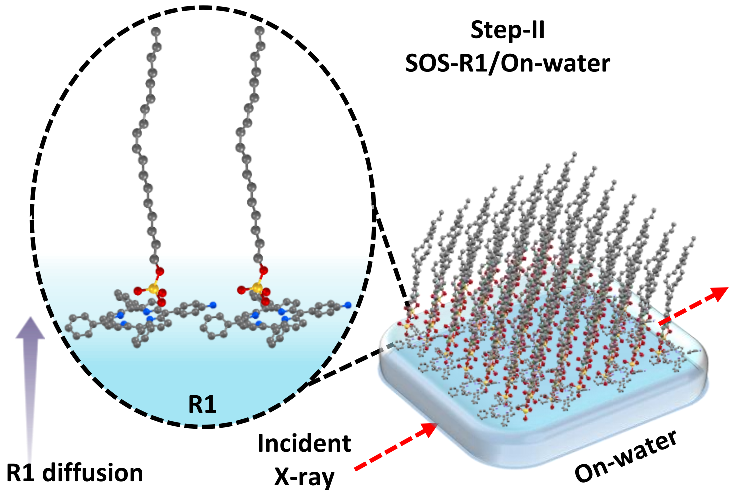

Supplementary Fig. 9. In-situ Grazing Incidence X-ray Diffraction (GIXD) on the water surface. Step-II, i.e., the pre-organization of R1 underneath the surfactant monolayer. Two hours after the injection of R1 into the water subphase and the subsequent diffusion of it, the self-assembled R1 structure underneath the surfactant monolayer.

Peak	(hk)	Q_{xy} [Å^{-1}]	d_{xy} [Å]
a	(10)	0.49	12.85
b	(01)	0.65	9.61
c	(1-1)	0.80	7.90
d	(11)	0.86	7.29
e	(20)	0.96	6.52
f	(2-1)	1.10	5.71
g	(21)	1.20	5.23
h	(30)	1.42	4.42
i	(12)	1.47	4.27
j	(3-1)	1.51	4.16

Supplementary Tab. 2. The corresponding measured GIXD scattering profile of **R1** structure underneath the SOS surfactant monolayer exhibited ten distinct and sharp diffraction peaks along with d_{xy} [Å] values.

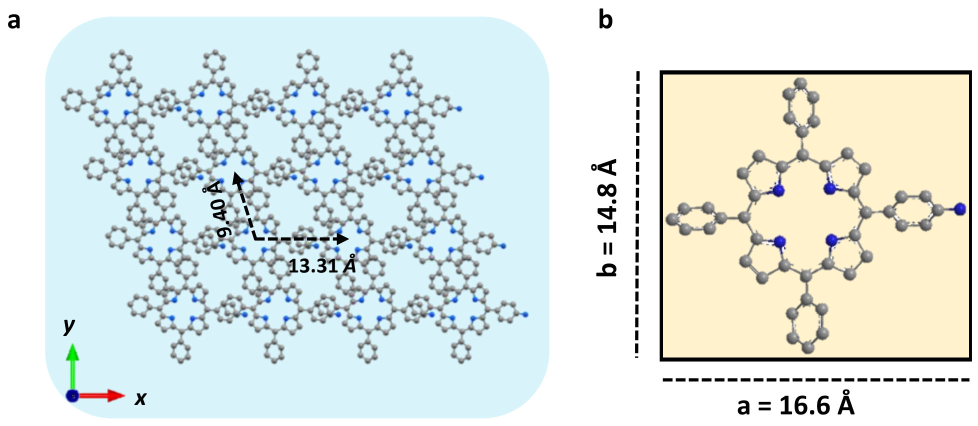

Supplementary Fig. 10. (a) The pre-organized **R1** structure on the water surface exhibits the lattice parameters a = 13.31 Å and b = 9.40 Å, which are significantly smaller than the (b) size of an individual **R1** molecule, a = 16.6 Å and b = 14.8 Å.

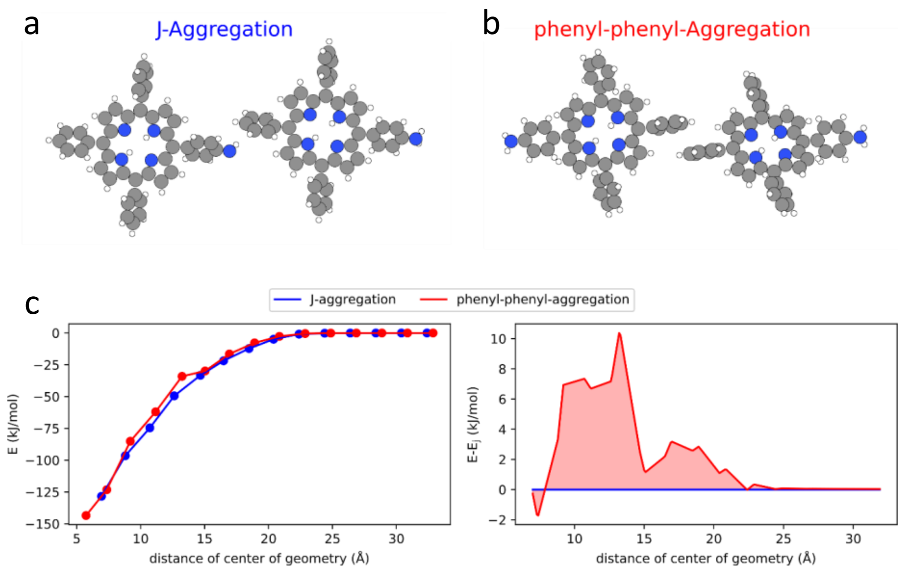

Supplementary Fig. 11. (a) J-aggregation pattern of two **R1** molecules. (b) phenyl-phenyl-aggregation pattern of two **R1** molecules. (c) Left panel: energy curve versus distance of center of geometry. E=0 kJ/mol is defined at maximum distance. Right panel: energy difference to the energy of the J-Aggregation (E_J). Energies in-between the calculated points were linearly interpolated.

Calculation of J-aggregation interaction energy

The energy of two **R1** molecules at different distances to each other (defined as the distance between their center of geometries) was calculated. We compared energies for the J-aggregation (NH_2 and phenyl) and the phenyl-phenyl-aggregation patterns with each other, as shown in (a) and (b), respectively.

The calculations were performed with density functional-based tight-binding theory (DFTB) with the software package[3] DFTB+. The 3ob-3-1 slater-koster parametrization was used[4]. Furthermore, a DFTB3-D3(BJ) , dispersion correction was applied[5] with the parameters a1=0.746, a2=4.191, s6=1.0 and s8=3.209. The maximum force component for the geometry relaxation with a conjugate gradient optimizer was chosen to be 1e-5 hartree/bohr and the SCC Tolerance criterion as 1e-07 e.

The distance between two molecules was constrained by fixing the positions of one outer atom in each molecule.

On the left panel in (c) depicts the energy curve for different distances is depicted. Here, it can already be seen that the π- π-aggregation is less favorable than the J-aggregation. This becomes clearer when the difference between energies is shown in the right panel. There it is visible that the π- π-aggregation is up to 10 kJ/mol less energetically favorable.

Computation of epitaxial alignments

With the lattice parameters for both the surfactant and the subphase determined by GIXD measurements and after finding that 1) the surfactant lattice does not change during the growth of the subphase and 2) the two lattices share in-plane peak positions, we also tested for possible epitaxial alignments between the two lattices. As explained in the main text, for the calculation of the epitaxial relationship we did not use the GIXD-derived unit cell of the surfactant with a = 5 Å, b = 7.48 Å, and γ = 89.96°, but instead one that is exactly half in size: a = 4.5 Å, b = 4.5 Å, and γ = 67.52°. This unit cell describes the lattice of the surfactant molecules without taking into consideration the azimuthal orientation of the molecules. The reason for doing so is grounded in our understanding that the **R1** assembly is driven by the electrostatic interaction between the anionic head group of the surfactant and the protonated **R1** molecule meaning the unit cell of the charged species is of importance and not the structural one. What the subphase **R1** molecules "see" is a lattice of charges in which the herringbone arrangement of the upper portion of the surfactant molecules, much of which is outside the water, likely plays a very small role. From this reduced, smaller surfactant unit cell and the **R1** lattice the areal density ratio of surfactant molecules (N_{SOS}) to **R1** molecules (N_{R1}) can be directly calculated from the inverse ratio of the respective unit cell areas: $N_{SOS} / N_{R1} = A_{R1} / A_S$ = 125.2 Å^2 / 18.7 Å^2 ≈ 6.7 ≈ 20:3. We can conclude from this that 3 **R1** molecules interact with 20 surfactant molecules., for this computational search we used a smaller, reduced unit cell for the surfactant lattice since we assume that the templating effect is a result of the electrical charges on the surfactant head groups. These charges however form this smaller unit cell of a = 4.5 Å, b = 4.5 Å, gamma = 67.52°. A software was used that analyzes all possible azimuthal alignments between the two lattices and searches for epitaxial configurations between the two within certain experimental tolerances (0.1 Å for the R1 lattice vectors). Algorithmically, this is achieved by calculating the algebraic 2x2-matrix that relates the substrate (here the surfactant SOS) to the grown-on-the-substrate lattice (here **R1**). With this matrix, the software then parses all possible azimuthal alignments between the two lattices while varying the subphase lattice within experimental error tolerances. For each investigated angle, the algorithm looks for matches between the two reciprocal lattices and classifies any found hypothetical epitaxial solutions according to[6]. This calculation detected

an epitaxial configuration between the two lattices where the **R1** lattice is rotated by -2.5° relative to the surfactant lattice (assuming 0° to mean that the two a vectors are collinear): a_R1=13.27Å, b_R1=9.49Å, gamma_R1=96.00°, epitaxy matrix [[-3.00 0.14],[1.00 -2.28]] with high confidence. This configuration is schematically shown in Supplementary Fig. 13. The fact that the matrix contains one column of integer values indicates that the arrangement is point-on-line coincident[7]. In this mode of epitaxy, the second lattice grows relative to the template lattice (surfactant) such that the two lattices coincide in one of their low-indexed crystallographic d-spacings, i.e. they are azimuthally aligned in such a way that the corresponding lattice lines, here d01 lattice lines, exactly coincide. It was previously shown that such an alignment is energetically advantageous compared to an arbitrary azimuthal alignment and therefore represents a type of epitaxy, despite being a more "subtle" type of lattice match compared to the typical commensurate inorganic epitaxy case that stems from the formation of covalent bonds.

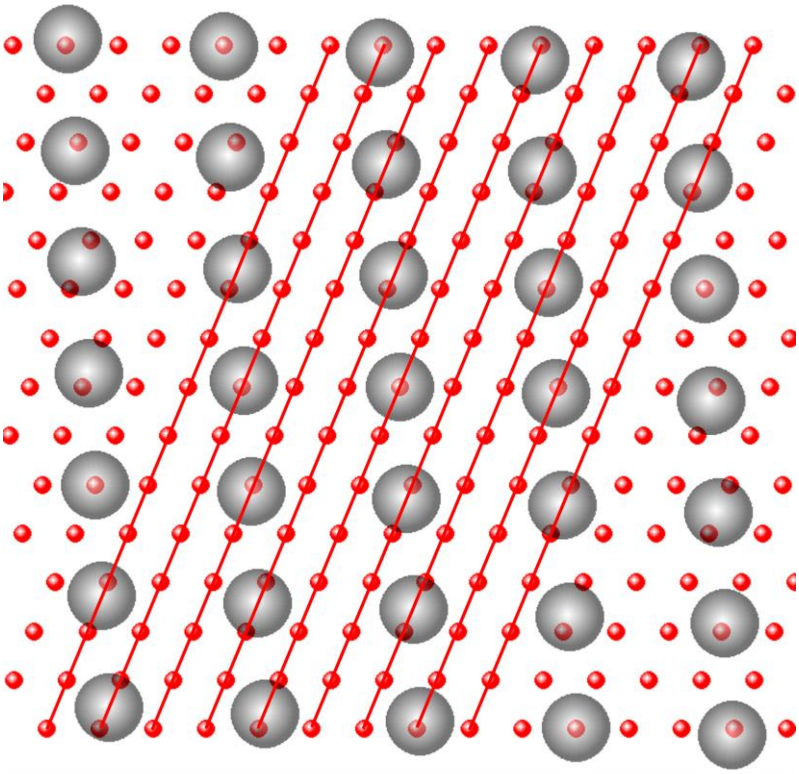

Supplementary Fig. 12. Schematic of the lattices of the surfactant SOS (red circles) and the epitaxial grown **R1** (gray circles). The red lines are guides for the eyes to better show the point-on-line coincidence of the two lattices.

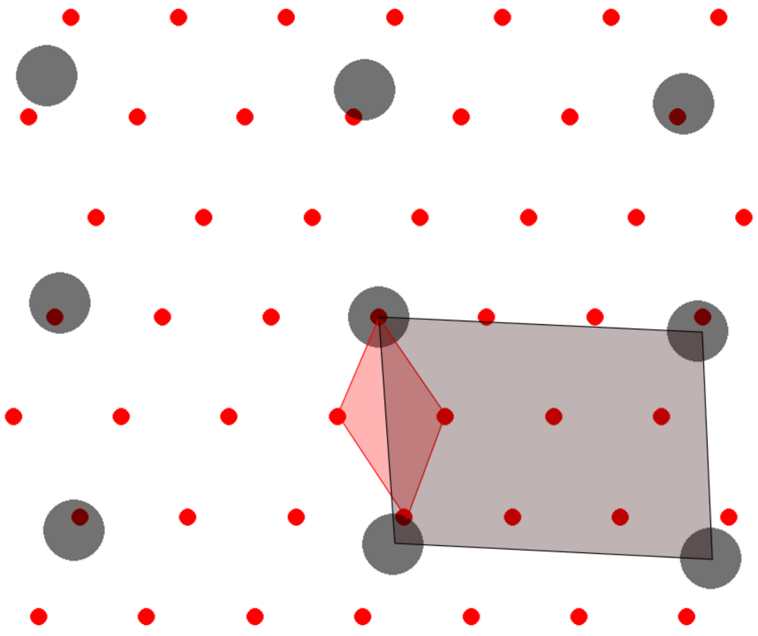

Supplementary Fig. 13. Schematic of the lattices of the surfactant SOS (red circles) and the epitaxial grown **R1** (gray circles). The filled areas represent the unit cells of the two materials used for calculating the epitaxial relationship.

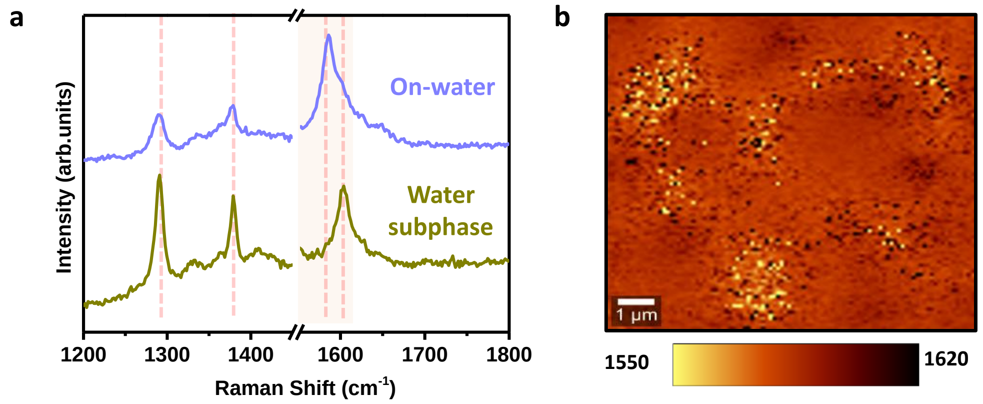

Supplementary Fig. 14. (a) All characteristic Raman peaks of **R1** were well-matched in both on-water surface film and water subphase, whereas a noticeable red shift in -NH_2 bending mode from 1620 to 1594 cm^{-1} was observed in the on-water surface film as compared to water subphase, demonstrating the strong interaction of the polarized- π binding motif leading to J-aggregation of **R1**. (b) 2D-Raman mapping revealed uniform coverage of the J-aggregated binding motif at the microscopic level (mapping-NH_2 bending mode).

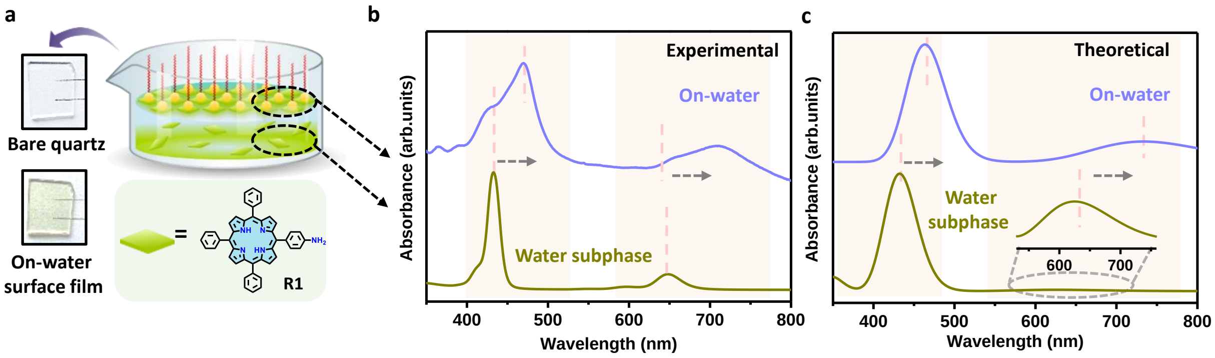

Supplementary Fig. 15. (a) Step-II is the most crucial step, which facilitates the pre-organization of **R1** underneath the surfactant monolayer (b) UV-Vis measurements were performed on both, the on-water surface film and the water subphase, after the completion of the step-II revealing the distinctive characteristics of both systems. A significant red shift in the Soret and Q-bands (peaked at 461 and 700 nm) in on-water surface film as compared to the water subphase (peaked at 430 and 650 nm) in UV-vis adsorption spectra, and these results are well supported by the (c) theoretical calculation.

We use density functional theory (DFT)[8,9] techniques to calculate the electronic properties of the porphyrin system studied in this article. We report the results using the wB97XD functional[10,11], and the 6-311+G(d) basis set as implemented in the Gaussian16 code[12].

TD-DFT[13,14] was used for the calculation of the linear excitations. We use water for the solvent calculations within the Polarizable Continuum Model (PCM) using the integral equation formalism variant (IEFPCM) [15-17], as implemented in Gaussian16. Gauss-View was used to visualize the electronic properties and vibrational modes of the studied systems. To understand and analyse the changes in the electronic environment of the dimer when laid on a solvent, additional calculation including the insertion of a few isolated water molecules were carried out. We optimize the position of 18 water molecules distributed around the dimer but restricting its geometry and defining a down and a top configuration (not shown).

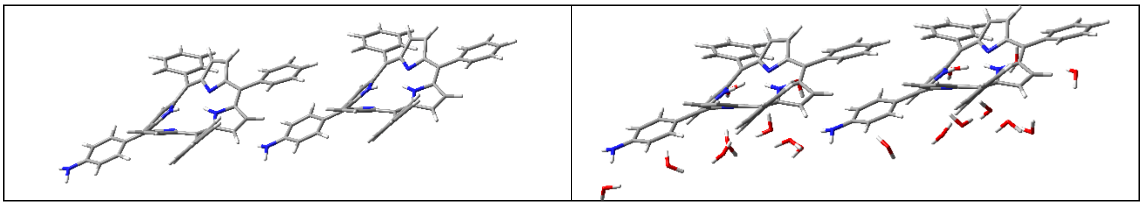

Supplementary Fig. 16. Structures of the dimer (left) and the dimer on-water (down configuration), which includes 18 explicit water molecules (right) for the calculations.

IR and UV/Vis spectra were calculated for the crystal structure of the **R1** and dimer system as seen in Supplementary Fig. 16 left. For completion, we optimize the structure of the **R1** and evaluate some optimization steps of the dimer structure. In the case of the **R1**, the curvature of the molecule is lost completely, while for the dimer structure a reduced curvature is (so far) retained, which we associate to interactions between the **R1** molecules, as the contour plots of the electrostatic potential surface (EPS) shows in Supplementary Fig. 17. The three-dimensional view of the EPS gives no detail information regarding the reactivity of the NH_2 group, therefore we use contour plots to show the charge distribution around the NH_2 group.

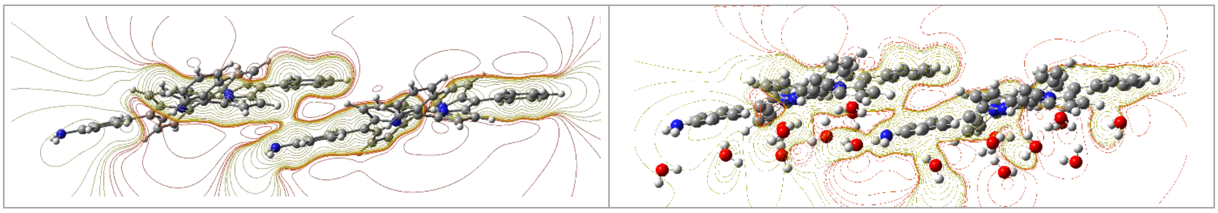

Supplementary Fig. 17. Contour plots of the electrostatic potential form the dimer structure (left) and from dimer structure with 18 water molecules below (right).

The contour plots in Supplementary Fig. 17 shows that there is some weak electronic interaction between the two molecular structures of the dimer, which is supported by the electronic distribution shown in the molecular orbitals (MO) HOMO and HOMO-1 (Supplementary Fig. 18). This interaction is present independent or not of having the molecule in vacuum (Supplementary Fig. 18) or supported on water.

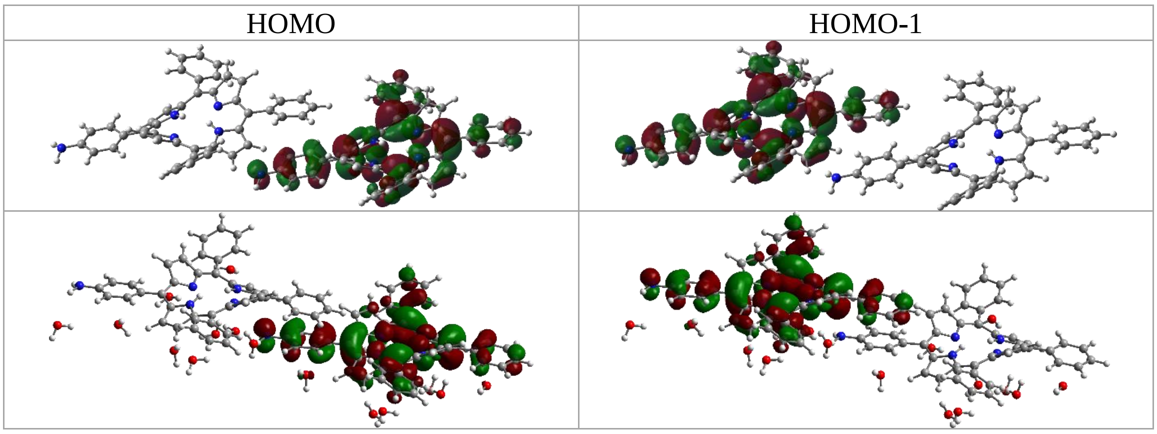

Supplementary Fig. 18. HOMO (left) and HOMO-1 (right) orbitals for the dimer structure (top), with explicit water molecules (bottom).

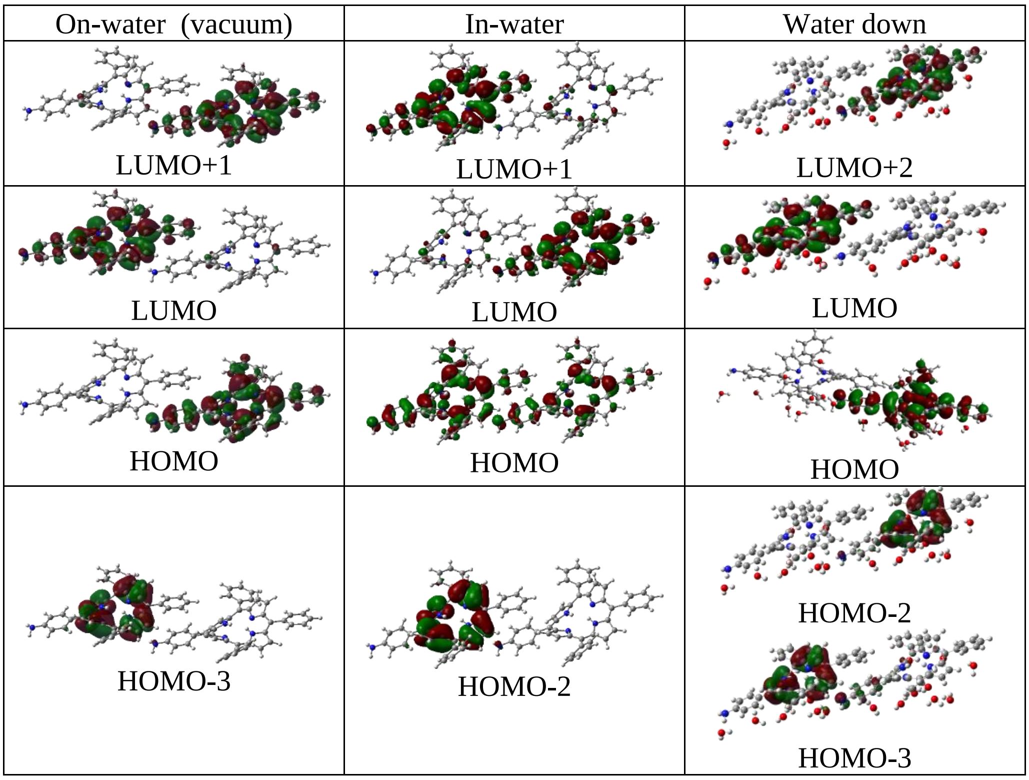

Supplementary Fig. 19. Molecular Orbitals

According to our calculations, the Soret band corresponds to an excitation from HOMO-3 to LUMO for the reaction on-water and from HOMO-2 to LUMO+1 for the reaction in-water. The Q-band shows

an excitation from HOMO to LUMO+1 and between HOMO and LUMO for the reactions on-water and in-water, respectively. Supplementary Fig. 19 shows the corresponding molecular orbitals.

Notice that the HOMO for the system in-water is delocalized, which means that this system shows less selectivity regarding the resistivity of the whole system compared to the HOMO of the on-water structure.

The EPS as well as the MOs supports the existence of a weak electronic interaction of the NH_2 group of the lower molecule with the above structure, independent of the presence of water molecules or not. The electronic environment of the system is only affected if we consider the reactivity of the system from above or below the system, but not in its intrinsic intermolecular electronic interactions, and not, therefore, in the stability of the system. The analysis of the UV spectra shows that the down configuration is close to the results obtained for the dimer structure in vacuum. We associate the small differences to small energy differences of the molecular orbitals involved in the excitation and we conclude thus that the role of the explicit water molecules considered is not strictly necessarily.

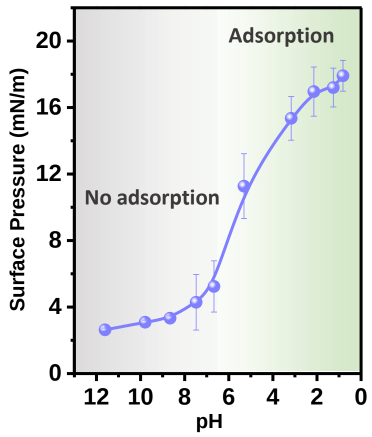

Supplementary Fig. 20. The impact of pH on the water surface film was further demonstrated by pH-dependent surface pressure measurements. As the pH became more acidic (from pH 4.6 to 0.9), the surface pressure increased linearly. However, with a basic pH (7 to 11), the surface pressure remained unchanged. This supports the previous UV-Vis results. The best pH for **R1** adsorption i.e., the interaction between protonated **R1** and the negatively charged head group of the surfactant was determined to be within the range of pH 3.8 to 0.9. The error bars illustrate the standard deviation derived from three replicated measurements.

In-situ on-water surface Sum Frequency Generation (SFG) spectroscopy

The SFG setup is schematically depicted in (Fig. 4a), where the visible (800 nm) and IR (2800-3600 cm^{-1}) beams are overlapped spatially and temporally at the sample position, and then the sum frequency is generated due to the centrosymmetric broken at the interface. In the present study, we used two SFG setup conditions, including homodyne SFG measurements for low-frequency region (1500-1800 cm^{-1}) and heterodyne (HD)-SFG measurements for high-frequency region (2800-3700 cm^{-1}). For homodyne measurements, the experiments were carried out with a femtosecond Ti: Sapphire amplified laser system (Spitfire Ace, Spectra-Physics, ~800 nm, ~40 fs, 1 kHz) with 5 W output power. Visible (13 µJ) and IR (5 µJ) incident angles were 36° and 41°, respectively. The generated SFG pulse was subsequently focused onto a spectrograph (Acton SP 300i, Princeton Instruments), and detected with an EM-CCD (Newton, Andor Technology). The spectra were collected in the *ssp* (denoting *s*-, *s*-, and *p*-polarized SFG, visible and IR, respectively) polarization combination. The spectra were normalized to the non-resonant signal taken from z-cut quartz after subtracting a background spectrum. For the HD SFG measurement, we show the detailed beam paths here, and put the description in the main text.

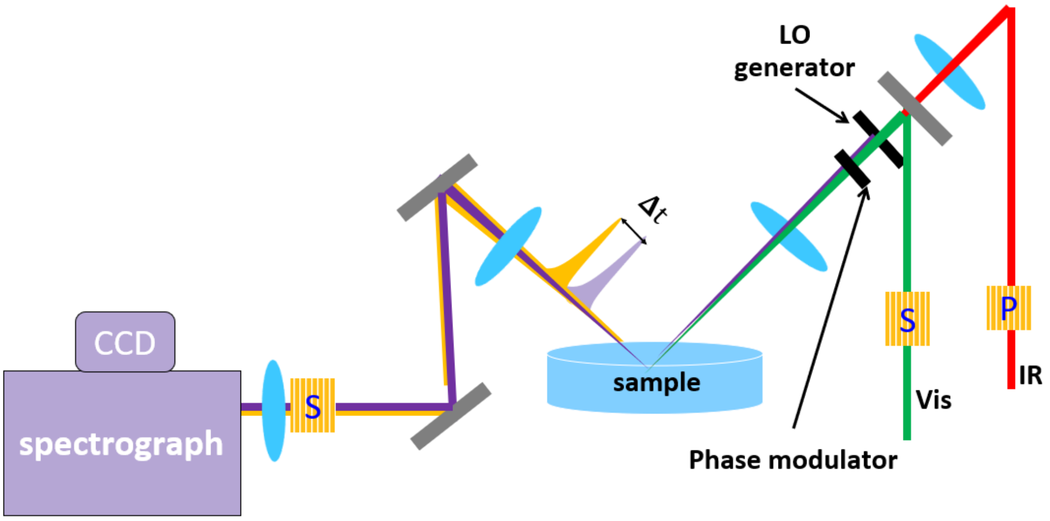

Supplementary Fig. 21. The Schematic of the collinear HD-SFG setup. The yellow box represents a polarizer, and the label marked as S or P represents the *s*- or *p*- polarization respectively.

Estimation of surface charge density from the SFG spectra under Gouy-Chapman model

Here we follow our previous work to estimate the surface charge density of SOS monolayer on the acidic solution (pH=1.8) surface[18]. At the negatively charged SOS-water interface, a SFG signal of water consists not only of the Stern layer contribution (${}^{(2)}_{R}(\omega)$ term) but also of the diffuse layer contribution (${}^{(3)}_{R}(\omega)$ term). The SFG signals are thus given by;

$$ {}^{(2)}_{\mathrm{eff}}(\omega,\sigma,c) = {}^{(2)}_{NR} + {}^{(2)}_{R}(\omega) + {}^{(3)}_{R}(\omega)\Phi(\sigma,c)\frac{\kappa(c)}{\kappa(c) - i\Delta k_z}, $$

(S1)

where $_{NR}^{(2)}$ is the non-resonant contribution. $_{R}^{(2)}(\omega)$ and $_{R}^{(3)}(\omega)$ are the second-order and third-order resonant contributions, respectively[19,20]. The mismatch of the wave-vectors, Δk_z, along the surface normal is $1/49$ (nm^{-1}) at ~3300 cm^{-1} region for IR frequency. $\Phi(\sigma, c)$ is the surface potential and is given by;

$$\Phi(\sigma, c) = \frac{2k_BT}{e_c}\sinh^{-1}\left(\frac{\sigma}{\sqrt{8000k_BTN_A\varepsilon_0\varepsilon_r c}}\right) \tag{S2}$$

in the Gouy-Chapman model. The inverse of the Debye length, $\kappa(c)$, is given by;[21]

$$\kappa(c) = \sqrt{\frac{2000e_c^2N_Ac}{\varepsilon_0\varepsilon_r k_BT}}, \tag{S3}$$

where ε_0, ε_r, k_B, T, e_c, and N_A denote the vacuum permittivity, the relative permittivity of water, the Boltzmann constant, temperature of the system, the elementary charge, and the Avogadro's number, respectively.

When a differential spectrum $\Delta_{\mathrm{eff}}^{(2)}(\omega, \sigma, c_1, c_2)$ is defined as

$$\Delta_{\mathrm{eff}}^{(2)}(\omega, \sigma, c_1, c_2) = \ _{\mathrm{eff}}^{(2)}(\omega, \sigma, c_2) - \ _{\mathrm{eff}}^{(2)}(\omega, \sigma, c_1), \tag{S4}$$

where the $_{\mathrm{eff}}^{(2)}(\omega, \sigma, c)$ is the measured SFG spectra at the ion concentration of c at the surface charge density σ, the $\Delta_{\mathrm{eff}}^{(2)}(\omega, \sigma, c_1, c_2)$ spectra can exclude the $^{(2)}$ contribution and thus can contain only the $_{R}^{(3)}$ term as;

$$\Delta_{\mathrm{eff}}^{(2)}(\omega, \sigma, c_1, c_2) = \ _{R}^{(3)}(\omega)\left(\frac{\Phi(\sigma, c_2)\kappa(c_2)}{\kappa(c_2) - i\Delta k_z} - \frac{\Phi(\sigma, c_1)\kappa(c_1)}{\kappa(c_1) - i\Delta k_z}\right). \tag{S5}$$

Furthermore, by computing $\frac{\Delta_{\mathrm{eff}}^{(2)}(\omega,\sigma,c_1,c_2)}{\Delta_{\mathrm{eff}}^{(2)}(\omega,\sigma,c_1,c_3)}$, one can obtain the ω-independent quantity;

$$f_1(\sigma, c_1, c_2, c_3) = \frac{\dfrac{\Phi(\sigma, c_3)\kappa(c_3)}{\kappa(c_3) - i\Delta k_z} - \dfrac{\Phi(\sigma, c_2)\kappa(c_2)}{\kappa(c_2) - i\Delta k_z}}{\dfrac{\Phi(\sigma, c_3)\kappa(c_3)}{\kappa(c_3) - i\Delta k_z} - \dfrac{\Phi(\sigma, c_1)\kappa(\ c_1)}{\kappa(c_1,) - i\Delta k_z}}. \tag{S6}$$

Since one can estimate the right side of eq. (S6) through the Gouy-Chapman model, one can obtain the surface charge density σ when the SFG spectra at the three different concentrations of c_1, c_2, c_3 are measured. By using this method, we obtain the surface charge density of SOS on the surface of HCl solution (pH=1.8) is -0.037±0.002 C/m^2, which is weaker compared to our previous value of SOS on the surface of H_2O. Considering the sulfate group with the pKa value of 2, it's reasonable to have a lower surface charge density at the acidic solution[22].

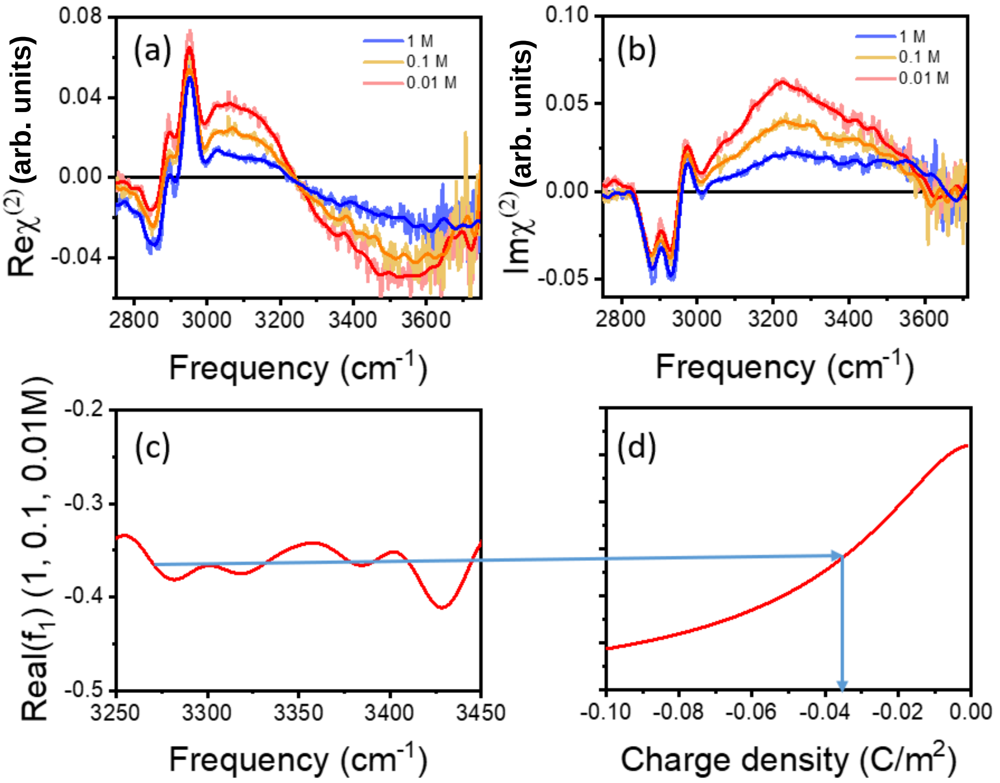

Supplementary Fig. 22. Surface charge estimation from the complex SFG responses.

(a-b) Complex SFG ($\mathrm{Im}_{\mathrm{eff}}^{(2)}(\omega, c)$ and $\mathrm{Re}_{\mathrm{eff}}^{(2)}(\omega, c)$) spectra obtained for various NaCl concentrations. (c-d) Experimentally obtained real part of f_1 at the SOS-HCl interface and calculated real part of the right-hand side of eq. (S7) as a function of the surface charge density at the SOS-HCl interface.

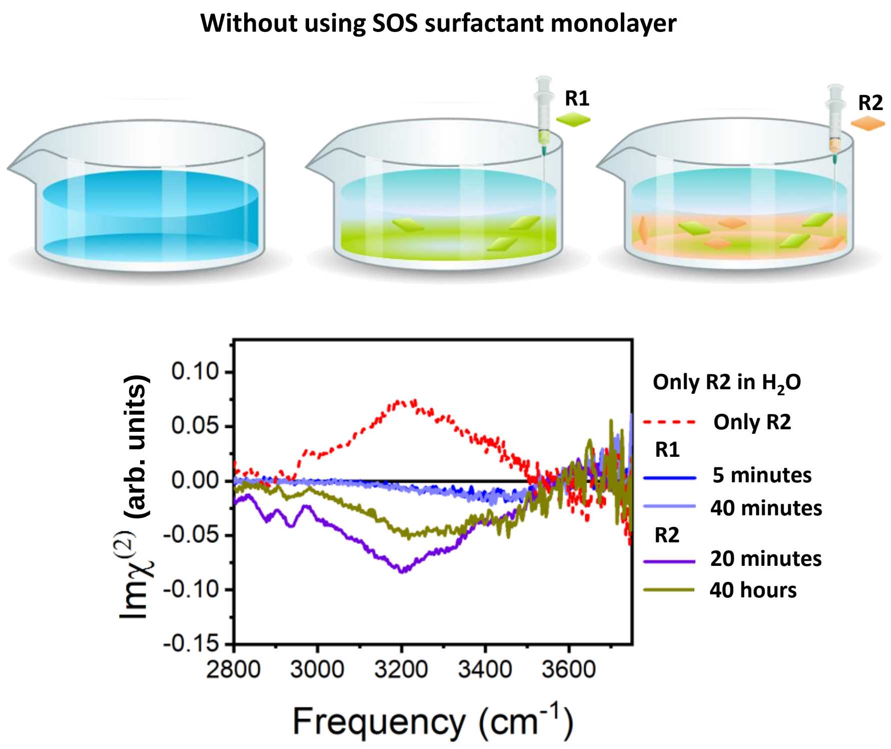

Supplementary Fig. 23. As a control experiment, we also did SFG measurements by injecting **R1** and **R2** into the water subphase without putting SOS surfactant on the water surface. The spectra show a negative contribution in the O-H stretch mode region, and we did not observe the C-H feature after injecting **R1**, indicating there is no **R1** adsorption at the interface in this condition. The O-H stretch mode becomes more negative, which means the surface becomes positively charged after injecting **R2**. The **R2** itself shows a positive O-H stretch mode feature, indicating that it is negatively charged at the interface (the red dotted line in Supplementary Fig. 23). The positively charged interface after adding **R2** to the **R1** solution can only come from the appearance of protonated **R1** at the interface. It means that **R1** is pushed to the surface after injecting **R2**, which can be due to the pH change in the solution, making **R1** less soluble. We did not observe the reaction product in this experimental condition, confirming the importance of the surface charge.

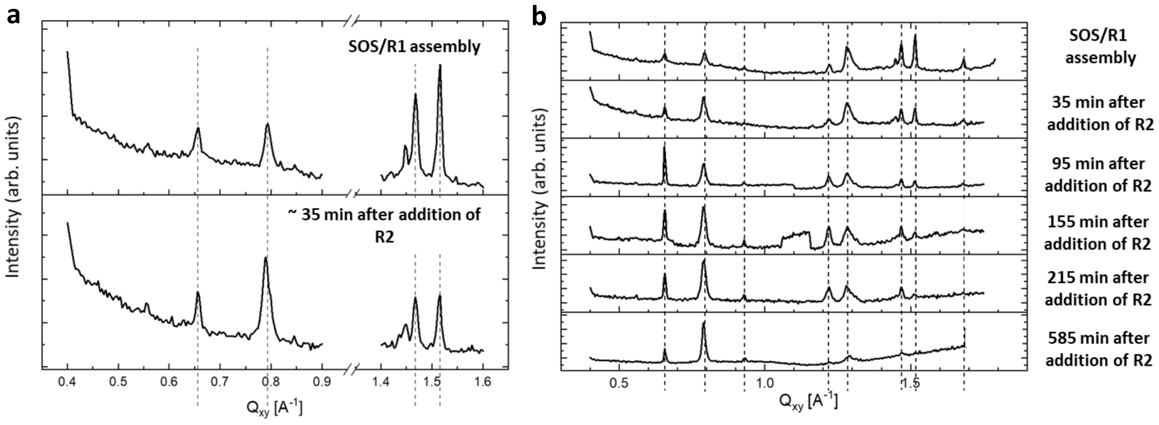

Supplementary Fig. 24. a) In-plane GIXD intensity profiles of the most characteristic peaks of the **SOS/R1** assembly (including the 110 peak of the surfactant) before (top) and 35 min after the addition of **R2** to the subphase. b) In-plane GIXD intensity profiles of the **SOS/R1** assembly before (top) and at various times after the addition of **R2**. In both figures, it can be seen that there is no change to the position of the diffraction peaks but that there is change to the relative peak intensities over time. This shows that the lattice of the assembly is maintained after addition of **R2** and we achieve a templated growth of **R2** beneath the assembly.

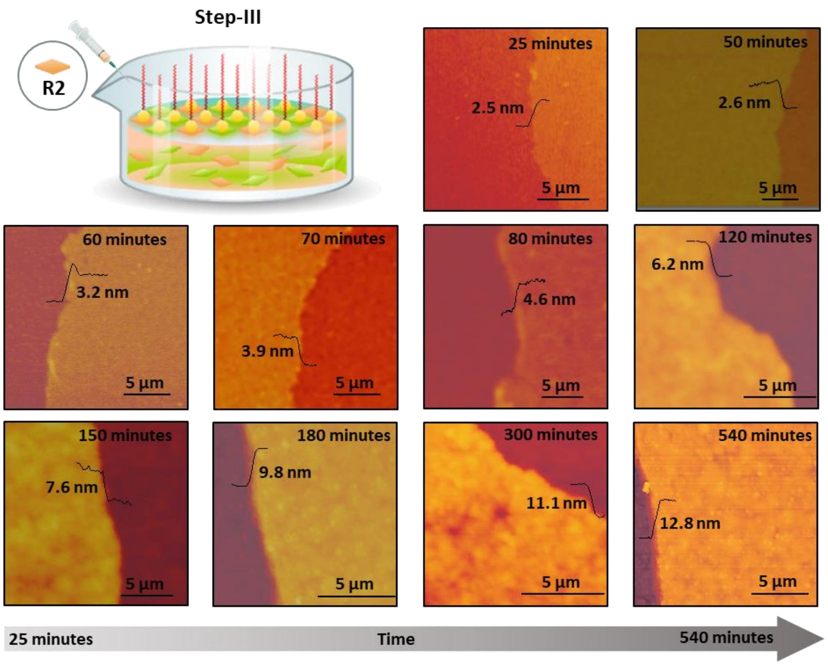

Supplementary Fig. 25. Time-dependent AFM analysis after adding **R2** into the water subphase.

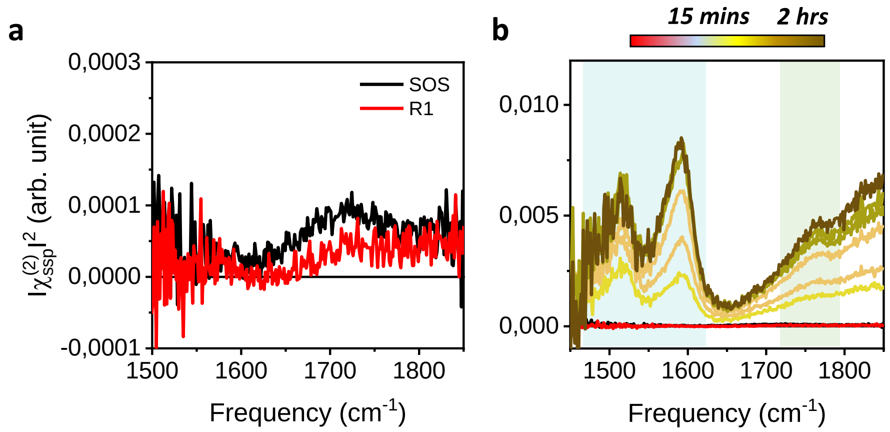

Supplementary Fig. 26. (a) Time evolutions of step-III i.e., after adding **R2** into the water subpahse solution. The C-N bend mode and the C=C and C=N stretch modes (1500–1850 cm^{-1}) regions are shown. The SFG spectrum at the SOS-water shows a weak H-O-H bending mode signal (black curve). After injecting **R1** into the subphase, there is no significant change in the spectrum compared to the spectrum of SOS-water (red curve). (b) After further injecting **R2** into the subphase, three peaks at ~1520, ~1590 and ~1760 cm^{-1} appear, which are assigned to the N–H in plane bending, the C–N stretching, and the C=O stretch mode respectively. Afterward, the three peaks were enhanced up to 100 minutes, indicating the stacking of the film.

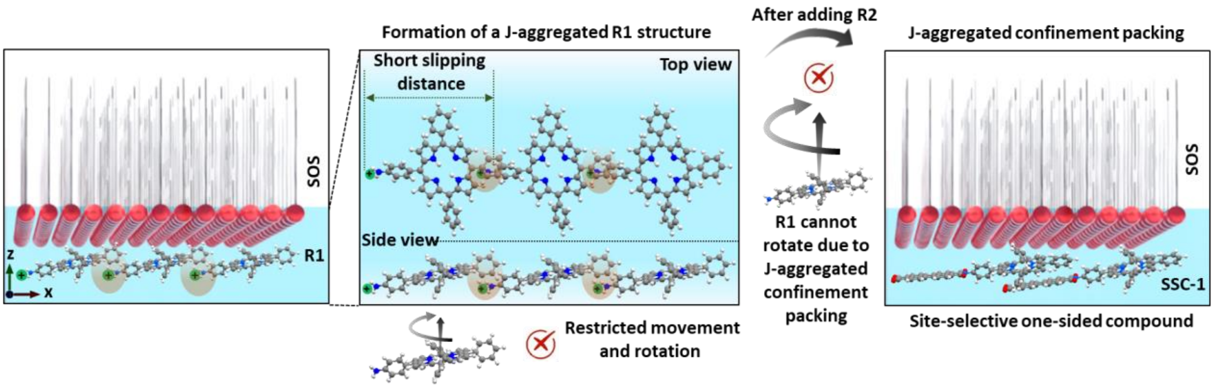

Supplementary Fig. 27. A schematic illustrating a site-selective chemical reaction on the water surface.

The surfactant monolayer on the water surface guides the epitaxial assembly of **R1** molecules into a well-defined J-aggregated structure and demonstrates strong polarized-π interaction within the **R1** structure, thereby restricting the movement or rotation of the J-aggregated **R1**. This constrained geometry of the porphyrin molecules facilitates the subsequent directional alignment of the **R2** reagent, leading to the selective formation of a one-sided imide product on the water surface.

References

1. Brown, P. J., Fox, A. G., Maslen, E. N., O'Keefe, M. A. & Willis, B. T. M. 6.1. Intensity of diffracted intensities. *International Tables for Crystallography C* **554** (2006).

2. Durbin, M. K. *et al.* Backbone orientational order in fatty acid monolayers at the air-water interface. *Phys. Rev. E.* **58**, 7686 (1998).

3. Hourahine, B. *et al.* DFTB+, a software package for efficient approximate density functional theory based atomistic simulations. *J. Chem. Phys.* **152**, 124101 (2020).

4. Gaus, M., Goez, A. & Elstner, M. Parametrization and benchmark of DFTB3 for organic molecules. *J. Chem. Theory Comput*. **9**, 338-354 (2013).

5. Kubillus, M., Kubar, T., Gaus, M., Rezac, J. & Elstner, M. Parameterization of the DFTB3 method for Br, Ca, Cl, F, I, K, and Na in organic and biological systems. *J. Chem. Theory Comput*. **11**, 332-342 (2015).

6. Mannsfeld, S. C. B. & Fritz, T. Advanced modelling of epitaxial ordering of organic layers on crystalline surfaces. *Mod. Phys. Lett. B.* **20**, 585-605 (2006).

7. Mannsfeld, S. B. & Fritz, T. Analysis of the substrate influence on the ordering of epitaxial molecular layers: The special case of point-on-line coincidence. *Phys. Rev. B.* **69**, 075416 (2004).

8. Hohenberg, P. & Kohn, W. Inhomogeneous electron gas. *Phys. Rev.* **136**, B864 (1964).

9. Kohn, W. & Sham, L. J. Self-consistent equations including exchange and correlation effects. *Phys. Rev.* **140**, A1133 (1965).

10. Grimme, S. Semiempirical GGA-type density functional constructed with a long-range dispersion correction. *J. Comput. Chem.* **27**, 1787-1799 (2006).

11. Chai, J.-D. & Head-Gordon, M. Long-range corrected hybrid density functionals with damped atom–atom dispersion corrections. *Phys. Chem. Chem. Phys.* **10**, 6615-6620 (2008).

12. Frisch, M. J. *et al.* Gaussian 16, Revision A. 03, Gaussian. *Inc., Wallingford CT* **3** (2016).

13. Gross, E. K. U. & Kohn, W. in *Advances in Quantum Chemistry* Vol. 21 (ed Per-Olov Löwdin) 255-291 (Academic Press, 1990).

14. Casida, M. E., Jamorski, C., Casida, K. C. & Salahub, D. R. Molecular excitation energies to high-lying bound states from time-dependent density-functional response theory: Characterization and correction of the time-dependent local density approximation ionization threshold. *J. Chem. Phys.* **108**, 4439-4449 (1998).

15. Miertuš, S., Scrocco, E. & Tomasi, J. Electrostatic interaction of a solute with a continuum. A direct utilizaion of AB initio molecular potentials for the prevision of solvent effects. *Chem. Phys*. **55**, 117-129 (1981).

16. Miertus, S. & Tomasi, J. Approximate evaluations of the electrostatic free energy and internal energy changes in solution processes. *Chem. Phys.* **65**, 239-245 (1982).

17. Pascual-ahuir, J.-L., Silla, E. & Tunon, I. GEPOL: An improved description of molecular surfaces. III. A new algorithm for the computation of a solvent-excluding surface. *J. Comput. Chem.* **15**, 1127-1138 (1994).

18. Seki, T. *et al.* Real-time study of on-water chemistry: Surfactant monolayer-assisted growth of a crystalline quasi-2D polymer. *Chem* **7**, 2758-2770 (2021).

19. Covert, P. A., Jena, K. C. & Hore, D. K. Throwing salt into the mix: Altering interfacial water structure by electrolyte addition. *J. Phys. Chem. Lett*. **5**, 143-148 (2014).

20. Wen, Y.-C. *et al.* Unveiling microscopic structures of charged water interfaces by surface-specific vibrational spectroscopy. *Phys. Rev. Lett.* **116**, 016101 (2016).

21. Bard, A. J., Faulkner, L. R. & White, H. S. *Electrochemical methods: fundamentals and applications*. (John Wiley & Sons, 2022).

22. Dean, D., Seog, J., Ortiz, C. & Grodzinsky, A. J. Molecular-level theoretical model for electrostatic interactions within polyelectrolyte brushes: applications to charged glycosaminoglycans. *Langmuir* **19**, 5526-5539 (2003).
